# The Usefulness of Combined Digital Dermatoscopy and Ultrasound with Colour Doppler in the Diagnosis of Skin Lesions

**DOI:** 10.3390/diagnostics15161992

**Published:** 2025-08-08

**Authors:** César Martins, Helena Pópulo, Paula Soares

**Affiliations:** 1 Médio Tejo Local Health Unit, 2350-750 Torres Novas, Portugal; wmed.medicos@sapo.pt; 2 Instituto de Investigação e Inovação em Saúde (i3S), Universidade do Porto, 4200-135 Porto, Portugal; psoares@ipatimup.pt; 3 Instituto de Patologia e Imunologia Molecular da Universidade do Porto (Ipatimup), 4200-135 Porto, Portugal; 4 Faculdade de Medicina, Universidade do Porto, 4200-319 Porto, Portugal

**Keywords:** digital dermatoscopy, cutaneous ultrasound, colour Doppler, skin cancer, vascularization

## Abstract

**Background**: Ultrasound and colour Doppler are adjuvant techniques widely used in clinical settings in obstetrics, cardiology, and others. Its use in dermatology is more incipient although it presents potential for clinical use namely in dermo-oncology. **Objective**: This study explores the usefulness of the combination of cutaneous ultrasound with Doppler after digital dermatoscopy in distinguishing between most common benign and malignant skin lesions, focusing on the importance of different vascular patterns. To streamline the diagnostic process, we propose a combined imaging workflow that integrates dermoscopic findings with vascular and structural data obtained via Doppler ultrasound. **Methods**: In total, 42 benign and malignant skin tumours were analysed in a population of 42 patients using a Fotofinder digital dermatoscopy device and a GE ultrasound machine with a high-frequency probe (20 MHz). Doppler was applied to assess lesion vascularization and identify distinct blood flow patterns. **Results**: Cutaneous ultrasound revealed that malignant lesions often exhibited intense and disorganized vascularization, while benign lesions displayed more ordered and peripheral blood flow patterns. In all of our cases, ultrasound with Doppler imaging clarified the uncertainties raised by dermatoscopy. **Conclusions**: The use of Doppler cutaneous ultrasound after digital dermatoscopy proved to be a valuable tool to aid the diagnosis in dermatology, as it improved the differential diagnosis between benign and malignant lesions, contributing to the establishment of the final diagnosis in the studied cases.

## 1. Introduction

Dermoscopy enhances visualization of skin structures using polarized and non-polarized visible light and magnification, facilitating detailed examination of surface features not easily discernible with the naked eye [1].

Internal light sources in the dermoscope illuminate the skin, enhancing contrast and clarity. Polarized light reduces surface glare, revealing deeper structures like blood vessels and pigmentation, while non-polarized light assesses surface characteristics such as scales and texture [2]. Dermoscopes typically offer magnification levels ranging from approximately 10× to 30×, addressing various diagnostic needs. Lower magnifications (around 10×) provide an overview of the lesion, while higher magnifications (up to 30×) allow for a more detailed examination of internal structures. Dermoscopy is routinely used in clinical practice to visualize skin microstructures, offering a resolution of approximately 10–30 μm and a penetration depth of up to 100 μm, enabling non-invasive analysis of both pigmented and non-pigmented lesions [3,4,5,6,7]. Dermoscopy is approved by both the FDA and EMA [8,9].

However, dermoscopically “false-positive” and “false-negative” tumours lead to unnecessary excisions or, in cancer lesions overlooking. The most frequent benign tumours that might acquire dermatoscopic characteristics suggestive of malignancy are seborrhoeic keratosis (including melanoacanthoma, irritated, clonal, and regressive), angioma (mainly thrombosed angioma and angiokeratoma), dermatofibroma, and naevi (Clark, Spitz, recurrent, combined, sclerosing). There are several dermatoscopic useful clues to recognize these tumours.

On the other hand, some malignant tumours might mimic benign ones and escape detection, namely melanoma (in situ, nevoid, spitzoid, verrucous, regressive, amelanotic), squamous cell carcinoma (mainly well-differentiated variants), or rarely basal cell carcinoma (non-pigmented variants). Also there are some dermatoscopic useful clues for the recognition of these tumours [10]. Therefore, management strategies should mainly focus on addressing the risk of dermoscopically false-negative tumours.

High-Frequency UltraSound (HFUS) utilizes sound waves, typically ranging from 20 to 50 MHz or higher, to generate detailed images of internal structures, particularly in skin cancer imaging. A transducer on the skin permits sound wave penetration, revealing tissue density and composition through echo analysis.

With a resolution of 40–200 μm and a 1–10 mm penetration depth, HFUS offers real-time visualization of skin layers and lesion characteristics, aiding in non-invasive assessment, diagnosis, and treatment planning for conditions such as skin cancer [11].

Cutaneous Doppler ultrasound is a non-invasive technique that uses high-frequency sound waves to visualize and measure blood flow in tissues. The Doppler effect allows for the observation of blood flow velocity and direction, providing crucial information about lesion vascularization [12]. The low-frequency probe (5–8 MHz) is used for the evaluation of deep blood vessels and is ideal for thicker skin lesions and for assessing subcutaneous vascularization [13]. On the other hand, the high-frequency probe (10–20 MHz) is suitable for superficial skin lesions and detailed assessment of cutaneous vascularization, providing a clearer image of superficial structures, facilitating identification of blood flow patterns. However, it has a limited depth of penetration, typically up to 2–3 cm [14].

The colour Doppler is an ultrasound imaging technique that visualizes blood flow within vessels or tissues by superimposing colour-coded flow information onto a greyscale image of anatomical structures. It provides a real-time, qualitative assessment of the direction and velocity of blood flow, allowing for the identification of flow abnormalities such as turbulence, blockages, or abnormal flow patterns. Colour Doppler assigns different colours to indicate the direction and relative velocity of blood flow. Typically, flow toward the transducer is shown in red, and flow away from the transducer is shown in blue. Brighter shades (e.g., lighter reds or blues) represent higher velocities, while darker shades indicate slower flow. This provides a continuous, real-time visual representation of blood flow dynamics superimposed on the anatomical structures. Colour Doppler is largely used for differentiating between benign and malignant lesions, facilitating identification of vascular patterns [15].

On the other hand, power Doppler is an advanced Doppler ultrasound technique that detects and visualizes blood flow based on the strength (amplitude) of the Doppler signal rather than on the direction or velocity of flow, as in traditional colour Doppler. It is highly sensitive to low-velocity or small-volume blood flow, making it ideal for assessing blood supply in tissues and organs in which traditional Doppler techniques might be less effective. It measures the strength of the Doppler signal, offering a more detailed visualization of blood vessel density and flow within the imaged area. This makes it particularly effective for identifying the presence of flow without concern for its direction. Due to its sensitivity, power Doppler is often used in oncology to assess tumour vascularity, namely neovascularization [13].

Recent studies have increasingly explored the integration of dermoscopy with high-frequency and Doppler ultrasound to improve diagnostic precision in ambiguous skin lesions. For instance, Ferrara et al. [13] demonstrated that colour Doppler imaging enhances the differentiation of melanoma from benign pigmented lesions by characterizing disorganized neovascular patterns. Despite these advancements, most studies have either focused exclusively on morphological features (via dermoscopy) or vascular patterns (via Doppler) in isolation. The eventual synergistic use of both modalities in routine dermatology consultations remains underexplored, particularly in prospective series using real-time clinical data. Furthermore, few publications provide a structured diagnostic algorithm integrating both surface morphology and internal vascular dynamics. This gap in practical guidance limits the adoption of Doppler imaging in dermatological practice, where time efficiency and diagnostic accuracy are crucial.

In the present study we report our results concerning the use of digital dermatoscopy and cutaneous ultrasound with colour Doppler in distinguishing between benign and malignant skin lesions, focusing on the relevance of vascular patterns. The integration of digital dermoscopy with Doppler ultrasound significantly improved diagnostic accuracy. Digital dermoscopy, which provides a detailed assessment of the lesion’s surface, including pigmentation patterns and superficial vascular structure, was complemented by Doppler ultrasound, which allowed for a deeper analysis of internal vascularization. This combined method not only facilitated the identification of vascular patterns associated with each type of lesion but also provided a comprehensive view of the lesion as a whole.

## 2. Materials and Methods

Patients and Sample: This study included 42 patients with various skin lesions who underwent evaluation in a dermatology clinic (Ethical approval No. 12/CECRI/2021).

The series comprised 23 women and 19 men, with a mean age of 58 years old (42−74 years). The patients included were selected sequentially over time, based on the need for additional diagnostic assessment.

The series includes cherry angioma (*n* = 6), seborrheic keratosis (*n* = 6), dermatofibroma (*n* = 6), melanocytic nevus (*n* = 6), melanoma (*n* = 6), basal cell carcinoma (*n* = 6), and squamous cell carcinoma (*n* = 6) (Table 1). All cases were identified based on clinical and dermatoscopic criteria and, when necessary, confirmed through histopathology.

Digital dermoscopy was performed with the FotoFinder Medical 1000 using both polarized and non-polarized light. Established diagnostic algorithms, including the ABCD rule, the 7-point checklist, and Menzies algorithm, were applied to characterize and differentiate the lesions.

All cases were examined in real time during clinical consultation, in a standardized setting with the patient seated under controlled lighting conditions. The dermatoscopic and ultrasound images were acquired and evaluated by a single experienced dermatology consultant (C.M.), with over five years of experience in cutaneous ultrasound and dermatoscopy. As this was a single-operator study, no blinding was applied.

During the ultrasound examination, a conductive gel was applied to ensure proper transmission of sound waves and minimize artifacts in the image. A GE LOGIC E ultrasound machine with a high-frequency probe (20 MHz) was used. Colour Doppler was applied to examine lesion vascularization. The probe was positioned over the lesion and gently moved to obtain detailed images of the structure and blood flow. Doppler was used to identify and map vascularization. The colour Doppler provided encoding of blood flow velocities and directions in colours, facilitating clear visualization of vascular patterns.

The vascularization patterns were classified as peripheral vascularization, centralized vascularization, and intense and disorganized vascularization. In peripheral vascularization, blood flow is more ordered and located at the edges of the lesion [16]; in the centralized pattern of vascularization, the blood flow is intense and disorganized at the centre of the lesion, indicating aggressive growth [17]; and a pattern of intense and disorganized vascularization is a strong indicator of malignant lesions [13].

## 3. Results

The clinic-pathologic and demographic data of the patients are summarized in Table 1.

In the digital dermatoscopic examination of normal skin, the epidermis appeared as a thin, homogeneous layer, while the dermis was visible with a well-organized architecture. Cherry angioma showed a pattern of small, round blood vessels (glomerular pattern). Seborrheic keratosis revealed central comedo-like (blackhead) openings, cerebriform pattern, and sometimes irregular vessels. Dermatofibroma often showed a pigmented reticular network, similar to melanocytic nevi and a central white scar-like area. A melanocytic nevus presented as a uniform network of pigment (reticular pattern), brownish or black dots or globules, mainly at the centre, with an absence of vascular structures. Melanoma showed heterogeneous pigmentation with multiple shades of brown, black, blue, and red and irregular or chaotic blood vessels, including dotted, linear, or arborizing vessels patterns. Basal cell carcinoma revealed telangiectatic blood vessels arranged in a branching or “tree-branch” pattern and sometimes, blue-grey ovoid nests. Squamous cell carcinoma mainly showed irregular keratinized areas; ulceration; variable pigmentation with a red, brown, or flesh-coloured appearance; and atypical blood vessels, including dotted, linear, or arborizing vessels.

In ultrasound examination, the normal epidermis appeared as a thin, homogeneous layer, while the dermis is visible with a well-organized architecture and normal blood vessels distributed in an orderly manner with adequate perfusion.

A summary of the vascularization patterns observed in benign and malignant lesions is provided in Table 2.

**Figure 1:**
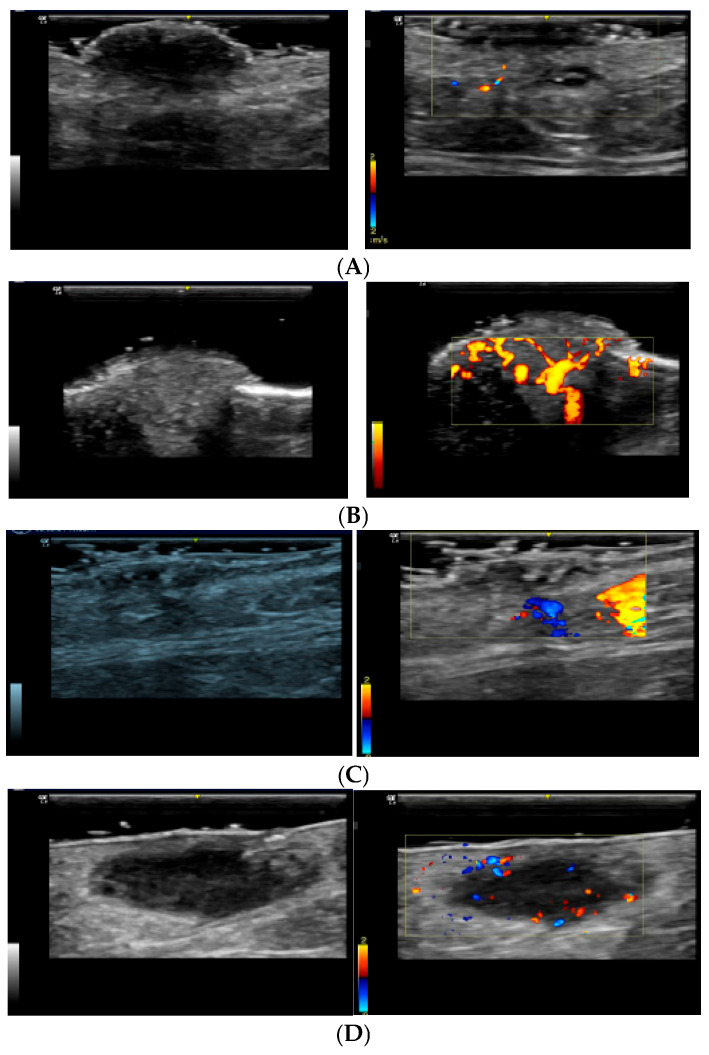
Colour Doppler ultrasound features of benign cutaneous lesions. (**A**) *Melanocytic nevus:* Peripheral vascularization with subtle, well-organized blood flow at the lesion’s edges. No central vascular signal detected, consistent with benign melanocytic proliferation. (**B**) *Cherry angioma:* Prominent superficial vascularization with dilated dermal vessels. Colour Doppler reveals intense, but highly organized, flow typical of benign vascular lesions. (**C**) *Seborrheic keratosis:* Low-flow peripheral vascularization with a well-circumscribed pattern. Absence of central chaotic signals supports a benign diagnosis. (**D**) *Dermatofibroma:* Peripheral vascular signals with little or no central flow. This avascular core and encapsulated appearance are characteristic of benign fibrohistiocytic lesions.

The vascularization patterns observed in the benign lesions are described below:

Melanocytic nevus: Typically presents with peripheral vascularization, with subtle blood flow at the edges of the lesion (Figure 1A). Colour Doppler usually reveals a well-defined pattern, indicating benignity [15].

Cherry angioma: Characterized by prominent superficial vascularization, with dilated vessels visible in the dermis (Figure 1B). Colour Doppler reveals a well-organized vascular pattern, not indicative of malignancy [15,18,19,20].

Seborrheic keratosis: Typically presents as a benign skin lesion with a variable degree of vascularization (Figure 1C). On colour Doppler, it shows a predominantly peripheral vascular pattern with a less intense flow compared with that in malignant lesions. The vascularization is generally well-defined and organized [15,21,22].

Dermatofibroma: Displays peripheral vascularization with little or no central vascularization (Figure 1D). This pattern suggests an encapsulated, benign lesion [17,23,24].

**Figure 2:**
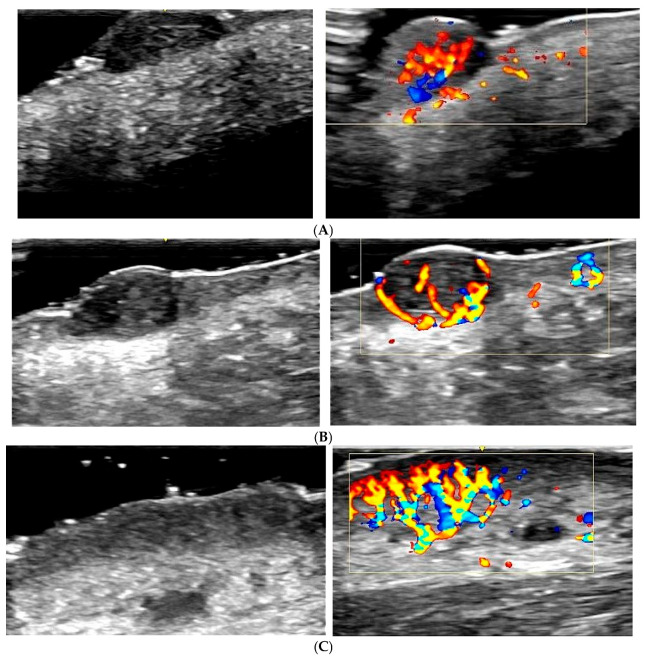
Colour Doppler ultrasound features of malignant cutaneous lesions. (**A**) *Melanoma:* Intense and disorganized vascular flow with irregular, chaotic patterns throughout the lesion. These findings are indicative of neovascularization and aggressive biological behaviour. (**B**) *Basal cell carcinoma (BCC):* Prominent peripheral vascularization with dilated vessels surrounding the lesion. Although less chaotic than melanoma, the increased vascular density supports malignancy. (**C**) *Squamous cell carcinoma (SCC):* High-velocity, intense vascular flow both centrally and peripherally. The combined pattern of disorganized vascularity and signal intensity is strongly suggestive of a malignant tumour.

The main vascularization patterns observed in malignant lesions were as follows:

Melanoma: Exhibits an intense and disorganized vascularization pattern, with irregular and chaotic blood flow, indicating neovascularization and aggressiveness (Figure 2A). Colour Doppler is crucial for identifying these features and differentiating melanoma from benign lesions [25,26].

Basal cell carcinoma: Shows pronounced peripheral vascularization, with dilated blood vessels around the lesion (Figure 2B). The peripheral vascularization pattern helps distinguish basal cell carcinoma from other lesions [16,27,28].

Squamous cell carcinoma: Demonstrates intense central and peripheral vascularization, with high-velocity blood flow (Figure 2C). Colour Doppler identifies these patterns as indicative of a malignant and aggressive tumour [18,28,29].

## 4. Discussion

The integration of digital dermoscopy with Doppler ultrasound represents a major advancement in the evaluation of skin lesions, providing a synergistic approach that enhances diagnostic accuracy and reliability [10,11,30]. This combined methodology addresses the diagnostic limitations of each technique when used independently, significantly improving the ability to differentiate between benign and malignant lesions [18,25,26,27,28].

Dermoscopy has been extensively used for morphological characterization of lesions, offering detailed visualization of pigmentation patterns, surface structures, and superficial vascularization [4,5,6]. However, it lacks the ability to assess deeper structures and intralesional vascularity, which can lead to false-positive and false-negative results [10]. Several studies have demonstrated that dermoscopy alone may not be sufficient for diagnosing equivocal lesions, leading to unnecessary excisions or overlooked malignancies [17,21,22]. This underscores the need for supplementary imaging techniques, such as Doppler ultrasound, to enhance diagnostic precision.

Doppler ultrasound offers unique advantages in dermatological imaging by assessing lesion vascularization, providing real-time visualization of blood flow dynamics, and identifying vascular patterns associated with malignancy [12,15]. Malignant lesions, particularly melanoma, basal cell carcinoma (BCC), and squamous cell carcinoma (SCC), exhibit increased vascularity with intense and disorganized blood flow, often indicative of angiogenesis and tumour progression [25,26,27,28]. The ability of Doppler ultrasound to detect these specific vascular patterns significantly improves diagnostic accuracy and aids in preoperative planning.

Several studies have validated the role of Doppler ultrasound in distinguishing between benign and malignant cutaneous lesions. Ferrara et al. [13] demonstrated that Doppler imaging reliably differentiates melanomas from benign pigmented lesions based on vascular flow characteristics. Similarly, Walter et al. [18] emphasized that Doppler ultrasound enhances specificity in identifying malignant lesions, reducing unnecessary biopsies and facilitating early detection. These findings highlight the clinical value of integrating Doppler ultrasound into routine dermatological assessments.

This study was designed as a preliminary observational assessment of combined dermatoscopy and Doppler ultrasound rather than as a diagnostic accuracy study. Therefore, formal statistical metrics such as sensitivity, specificity, or *p*-values were not calculated. Future prospective studies with larger sample sizes should aim to provide such statistical validation.

In our study, the combined use of dermoscopy and Doppler ultrasound proved to be particularly effective in clarifying diagnostic uncertainties. Lesions that exhibited ambiguous dermoscopic features were further evaluated using Doppler ultrasound, allowing for better characterization based on vascular patterns. This approach was instrumental in reducing false positives, preventing unnecessary excisions of benign lesions such as dermatofibromas and seborrheic keratoses [15,17,21,22]. Furthermore, in all cases of confirmed malignancy, histopathological findings were consistent with Doppler ultrasound assessments, reinforcing its diagnostic reliability.

This study is limited by the small sample size (42 lesions), which may affect the generalizability of the findings. However, the results offer preliminary but relevant insight into the potential diagnostic value of combining digital dermatoscopy with colour Doppler ultrasound in clinical practice. Future studies with larger patient cohorts are warranted to validate and expand upon these findings.

Proposed Practical Recommendations for Clinical Use

Based on our findings, we propose that the combined use of digital dermoscopy and Doppler ultrasound be implemented in dermatology consultations under the following indications:

- When dermoscopic features are equivocal, such as atypical pigment patterns without clear criteria for excision;
- In nodular or vascular-appearing lesions, where depth and vascular flow patterns may guide urgency of referral or excision;
- To reduce unnecessary excisions in benign-appearing lesions such as dermatofibromas or seborrheic keratoses, particularly in cosmetically sensitive areas;
- To assist in pre-surgical planning, helping define lesion depth, vascularity, and potential involvement of adjacent structures.

In daily practice, this method requires minimal adaptation, as both dermoscopy and Doppler ultrasound are non-invasive, well-tolerated, and rapidly executable techniques. The diagnostic algorithm we propose offers a practical flowchart to guide dermatologists:

Step 1: Initial Dermoscopic Examination: Assess lesion morphology, pigmentation, and superficial vascular patterns;

Step 2: Doppler Ultrasound Assessment: Evaluate lesion depth, vascularization, and blood flow dynamics.

Low or absent vascular flow → Suggests benign lesions (e.g., seborrheic keratosis, dermatofibroma, melanocytic nevus) [15,19,20,21].

Moderate vascular flow → Suggests benign vascular lesions (e.g., cherry angiomas) [15,19,20].

High-intensity or chaotic vascular flow → Strongly suggests malignancy (e.g., melanoma, SCC, BCC), requiring biopsy for confirmation [25,26,27,28,29,31].

This structured diagnostic workflow enhances the sensitivity and specificity of skin lesion assessment, optimizing patient management by reducing unnecessary interventions and enabling timely treatment of malignant lesions. Additionally, the integration of Doppler ultrasound into routine dermatology practice has significant implications for non-invasive cancer screening, offering a cost-effective and efficient alternative to histopathological examination in selected cases [30].

This study was designed as a single-operator, qualitative assessment reflecting real-time diagnostic practice. Therefore, no interobserver variability analysis was conducted. The aim was to explore practical diagnostic patterns rather than to validate a quantitative diagnostic tool.

While we acknowledge that colour Doppler ultrasound in dermatology remains a largely qualitative tool, we would like to emphasize that the most diagnostically relevant feature is not the absolute flow velocity or vessel density but rather the distribution and localization of vascular signals within the lesion—whether central, peripheral, disorganized, or absent.

In all of our cases, ultrasound with Doppler imaging clarified uncertainties raised by dermatoscopy. This combination of techniques proved invaluable, as it provided a more comprehensive understanding of the vascular characteristics of the lesions.

While previous studies have explored the individual value of Doppler ultrasound or its integration with dermoscopy, our study introduces a structured and reproducible diagnostic workflow that combines both modalities in a systematic manner, reinforced by histopathological validation. This dual approach demonstrated real-world utility in improving diagnostic clarity and reducing unnecessary surgical excisions.

The integration of these two imaging modalities significantly enhances diagnostic accuracy by combining surface morphological assessment with real-time vascular mapping. This dual approach not only improves the sensitivity and specificity of skin lesion diagnosis but also reduces unnecessary biopsies for benign lesions and facilitates early detection of malignancies.

## 5. Conclusions

High-frequency cutaneous ultrasound with colour Doppler is a revolutionary tool in dermatology, allowing detailed visualization of lesion vascularization. The ability to differentiate between benign and malignant vascularization patterns is crucial for accurate diagnosis and appropriate treatment. The integration of advanced imaging technology, such as Doppler, enhances the ability to identify and manage skin lesions effectively.

As technological advancements continue to refine imaging modalities, future studies should focus on the development of AI-driven diagnostic algorithms that incorporate dermoscopic and Doppler ultrasound data. Machine learning models trained on extensive datasets could further improve lesion classification, minimizing observer variability and enhancing diagnostic consistency. Moreover, prospective multicenter studies are needed to validate the long-term benefits of this combined approach in clinical practice, particularly in large-scale screening programs.

A recent survey by Diluiso et al. [32] demonstrated that, although 72% of surveyed plastic surgeons in Europe were aware of high-frequency ultrasonography, only 34% employed it routinely. This discrepancy between knowledge and clinical implementation highlights the barriers that clinicians face—both cognitive and structural—when adopting novel diagnostic technologies.

Our study addresses this challenge by proposing a streamlined, duplex imaging protocol combining dermoscopy and Doppler ultrasound, operable in real time by a single dermatologist. By demonstrating its diagnostic reliability and workflow simplicity, we offer a scalable approach that may promote wider adoption of vascular imaging in routine dermatological practice, helping to close the gap identified by Diluiso et al. [32].

## Figures and Tables

**Table 1:** Characteristics of patients.

Clinical Diagnosis	*n*	Mean Age (Range Years)	Gender (Women/Men)
Melanocytic Nevus	6	42 (35–50)	4/2
Cherry Angioma	6	51 (45–58)	3/3
Seborrheic Keratosis	6	69 (60–78)	5/1
Dermatofibroma	6	54 (46–62)	4/2
Melanoma	6	45 (38–52)	3/3
Basal Cell Carcinoma	6	71 (65–78)	3/3
Squamous Cell Carcinoma	6	74 (68–81)	1/5

**Table 2:** Vascularization patterns in cutaneous lesions.

Type of Lesion	Vascularization Pattern
Melanocytic Nevus	Peripheral, ordered vascularization
Cherry Angioma	Prominent superficial vascularization
Seborrheic Keratosis	Peripheral, low-intensity, organized pattern
Dermatofibroma	Encapsulated peripheral vascularization, minimal central flow
Melanoma	Intense, disorganized blood flow, chaotic pattern
Basal Cell Carcinoma	Pronounced peripheral vascularization
Squamous Cell Carcinoma	Intense central and peripheral vascularization, high velocity

## Data Availability

Data supporting the findings of this study are available from the corresponding author upon reasonable request.
